# Supplementary material for: Inflammatory factors and risk of lung adenocarcinoma: a Mendelian randomization study mediated by blood metabolites
Source: Front Endocrinol (Lausanne). 2024 Aug 27;15:1446863. doi: 10.3389/fendo.2024.1446863 (PMC11384989; doi:10.3389/fendo.2024.1446863)
Supplement: Supplementary file 1 [file DataSheet1.zip › supplementary files/S2/GCST90200922/sensitivity-analysis.pdf]

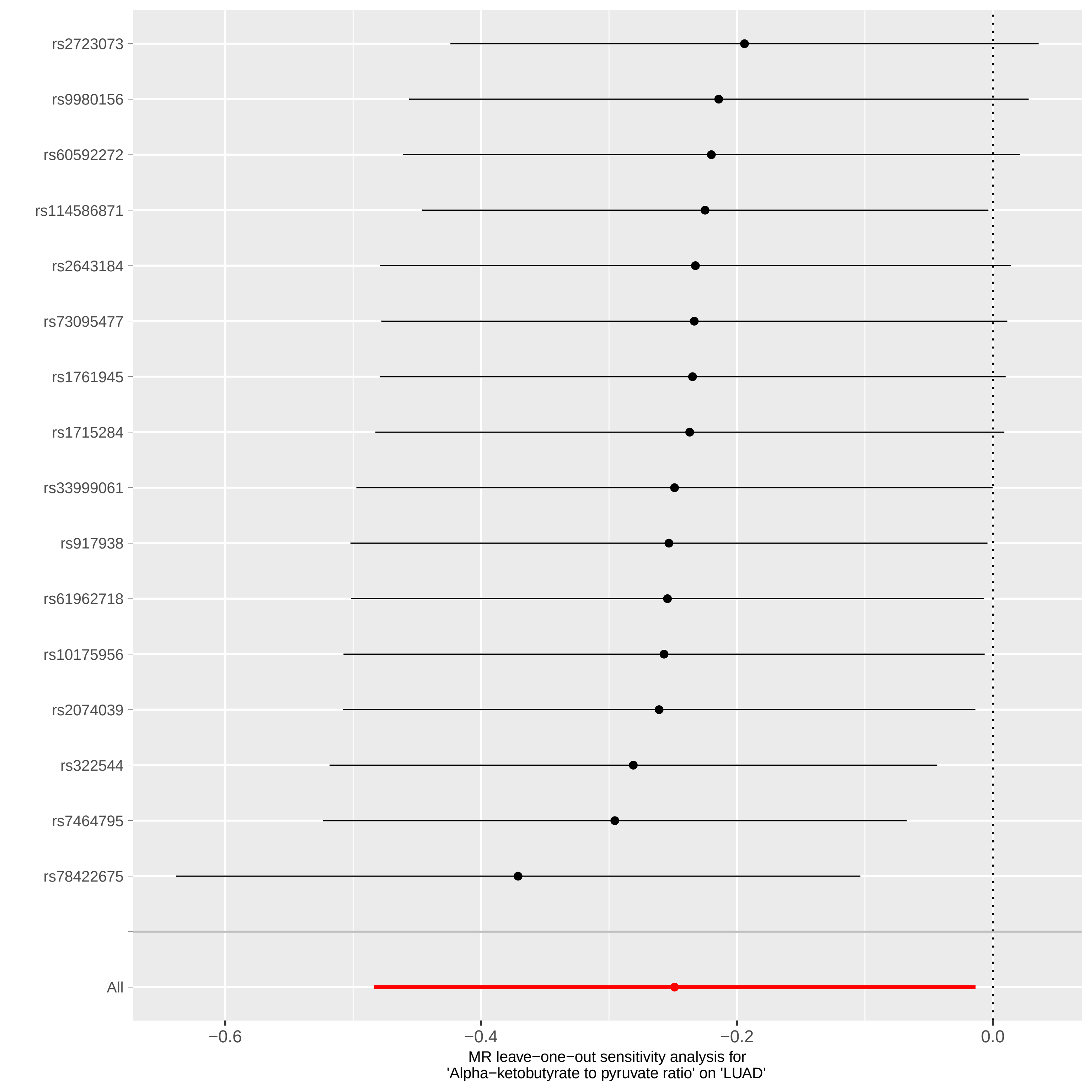
rs2723073
rs9980156
rs60592272
rs114586871
rs2643184
rs73095477
rs1761945
rs1715284
rs33999061
rs917938
rs61962718
rs10175956
rs2074039
rs322544
rs7464795
rs78422675
All
−0.6
−0.4
−0.2
0.0
MR leave−one−out sensitivity analysis for
'Alpha−ketobutyrate to pyruvate ratio' on 'LUAD'
